# Utility of Lymphoblastoid Cell Lines for Induced Pluripotent Stem Cell Generation

**DOI:** 10.1155/2016/2349261

**Published:** 2016-06-07

**Authors:** Satish Kumar, Joanne E. Curran, David C. Glahn, John Blangero

**Affiliations:** ^1^South Texas Diabetes and Obesity Institute, University of Texas Rio Grande Valley School of Medicine, Brownsville, TX, USA; ^2^Olin Neuropsychiatry Research Center, The Institute of Living, Hartford, CT, USA; ^3^Department of Psychiatry, Yale University School of Medicine, New Haven, CT, USA

## Abstract

A large number of EBV immortalized LCLs have been generated and maintained in genetic/epidemiological studies as a perpetual source of DNA and as a surrogate* in vitro* cell model. Recent successes in reprograming LCLs into iPSCs have paved the way for generating more relevant* in vitro* disease models using this existing bioresource. However, the overall reprogramming efficiency and success rate remain poor and very little is known about the mechanistic changes that take place at the transcriptome and cellular functional level during LCL-to-iPSC reprogramming. Here, we report a new optimized LCL-to-iPSC reprogramming protocol using episomal plasmids encoding pluripotency transcription factors and mouse p53DD (p53 carboxy-terminal dominant-negative fragment) and commercially available reprogramming media. We achieved a consistently high reprogramming efficiency and 100% success rate using this optimized protocol. Further, we investigated the transcriptional changes in mRNA and miRNA levels, using FC-abs ≥ 2.0 and FDR ≤ 0.05 cutoffs; 5,228 mRNAs and 77 miRNAs were differentially expressed during LCL-to-iPSC reprogramming. The functional enrichment analysis of the upregulated genes and activation of human pluripotency pathways in the reprogrammed iPSCs showed that the generated iPSCs possess transcriptional and functional profiles very similar to those of human ESCs.

## 1. Introduction

Epstein-Barr virus (EBV) immortalized lymphoblastoid cell lines (LCLs) have been routinely used as surrogate* in vitro* cell models for various human primary tissues to study genetic influence on disease traits [1], drug response [2–5], and gene regulation [6, 7]. Large numbers of stored human LCLs have been collected in genetic/epidemiological studies as a perpetual source of DNA. For example, the NIMH Repository and Genomic Resource alone currently stores over 184,000 LCLs [8] for sharing with investigators of mental disorders. LCLs have clearly been a convenient and useful model in the absence of primary tissue accessibility and therefore widely banked to study a variety of human diseases, including complex genetic disorders. However, their ability to faithfully recapitulate the specific regulatory properties of the donor's primary tissue has always been debated. A number of studies have characterized differences in gene regulatory phenotypes between LCLs and primary tissues [9–12]. An alternative to the LCL model is the stem cell based system, which carries the potential to model the tissue specific physiology through the use of differentiation protocols to generate specific cell/tissue types. The invention of the induced pluripotent stem cell (iPSC) technology allowed patient-specific, mature somatic cells to be converted into an unlimited supply of human pluripotent stem cells (hPSCs) [13, 14]. However, dermal fibroblasts isolated from skin biopsies by and large remain the material of choice for reprogramming experiments due to the low reprogramming efficiency of other cell types including LCLs. There exists a rich bioresource of numerous LCL repositories generated from wide array of patients, many of them with extensive genotypic and phenotypic data already generated; however, these remain severely underused for this purpose. Recent developments in the reprogramming protocol have made it possible to reprogram LCLs into iPSCs [15, 16] but, due to the heterogeneity of the starting material and complexity of the optimization of various media components and reprogramming factors, the reprogramming efficiency and overall success rate remain poor. We have been able to optimize a very efficient reprogramming protocol using stored LCLs, episomes available from a public source, and commercially available media, achieving 100 percent success rate and high reprogramming efficiency (see Materials and Methods for details).

The molecular events leading to the maintenance of pluripotency in embryonic stem cells (ESCs) and reacquisition of a stem-like state in iPSCs during somatic reprogramming represent mechanistically distinct processes that converge on a set of remarkably similar transcriptional events that underpin the pluripotent state [17]. Both ESCs and iPSCs depend on fundamental transcription frameworks that are governed by a common set of “core” stem cell-specific transcription factors, namely, OCT4, SOX2, and NANOG [18]. These activators in turn collaborate with both ubiquitous and cell type-specific transcription factors to orchestrate complex gene expression programs that give stem cells the unique ability to safeguard stemness while remaining poised to execute a broad range of developmental programs that drive lineage specification [19–22].

Although some success in developing a more efficient LCL-to-iPSC reprogramming protocol has been achieved [23], little is known about the mechanistic changes that take place at the transcriptome and cellular functional level during reprogramming of LCLs into iPSCs.

In this study, we sequenced six LCLs and their reprogrammed iPSCs for miRNome (microRNA/miRNA) and transcriptome (mRNA). We analyzed these dynamic datasets, aiming at identifying the functional changes at the global gene expression levels during LCLs-to-iPSC reprogramming process. A differential gene expression analysis was performed between LCLs and generated iPSC in combination with functional annotations and Ingenuity® Pathway Analysis (IPA).

## 2. Materials and Methods

We have a rich resource of LCLs established using the peripheral blood mononuclear cells (PBMCs) collected from more than 1400 Mexican American participants of our San Antonio Family Heart Study (SAFHS). Whole genome sequence data and extensive phenotype data for common complex human diseases are available for most of these SAFHS participants. Our large, well characterized LCL resource provides a unique opportunity to generate pluripotent stem cells from any of these individuals in the context of their own particular genetic identity for disease modeling, particularly by differentiating specific cell/tissue type from generated iPSC to experimentally test the hypotheses developed by statistical genetics methods.

The six human lymphoblastoid cell lines used in this study were previously established* in vitro* from blood samples of our SAFHS Mexican American participants from whom appropriate written consent was obtained. The six LCLs were deidentified and labeled as LCL-1 through LCL-6. The donors of LCL-1 and LCL-3 were diagnosed with sporadic Parkinson's disease. The donors of LCL-2, LCL-4, LCL-5, and LCL-6 were healthy. The donors of LCL-3 and LCL-4 were first-degree relatives whereas all other donors were unrelated. Institutional Review Board of the University of Texas Health Science Center at San Antonio (San Antonio, TX) approved all protocols used in this study.

### 2.1. Cell Culture

The six LCLs were thawed from the SAFHS cell line repository. The thawed cell lines were cultured in RPMI 1640 complete media (i.e., RPMI 1640 media containing 15% heat inactivated fetal bovine serum, 1% MEM nonessential amino acids, 1 mM sodium pyruvate, and 10 mM HEPES buffer, all from Life Technologies) at 37°C, 5% CO_2_, and atmospheric O_2_ for 1-2 passages to obtain the appropriate number of viable cells.

### 2.2. iPSC Reprogramming and Validation

**Figure 1:**
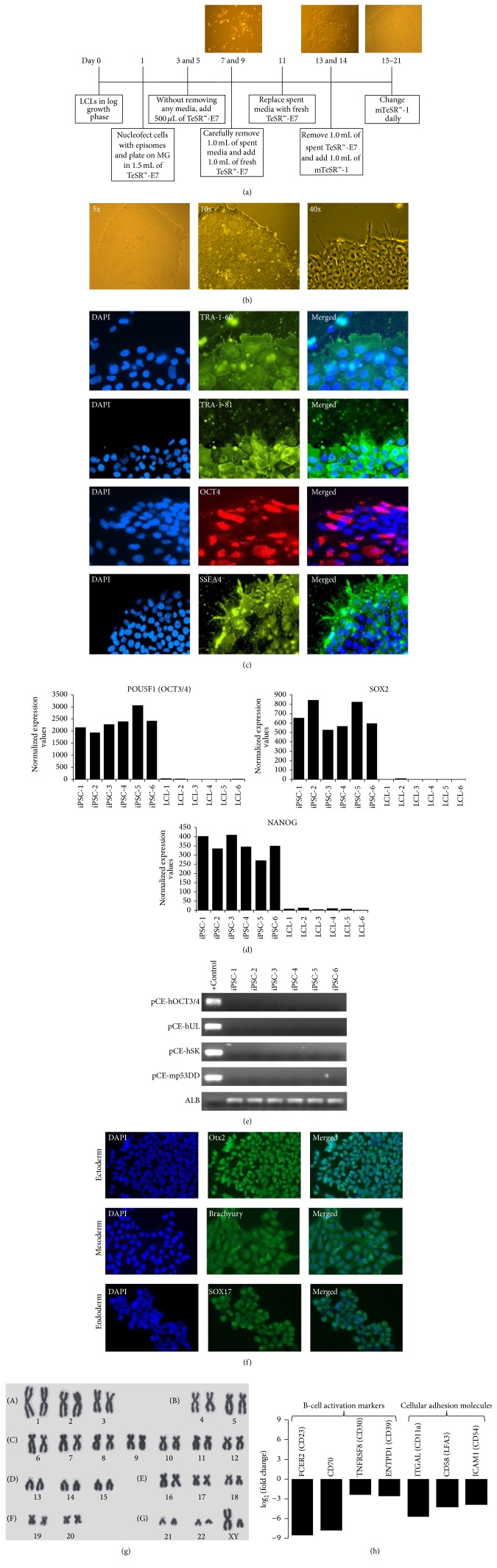
LCL-to-iPSC reprogramming and characterization. (a) Schematic diagram of LCL-to-iPSC reprogramming. (b) Morphology of a reprogrammed iPSC colony at 5x, 10x, and 40x original magnifications, respectively. (c) Immunocytochemistry analysis of generated iPSCs showing expression of pluripotency markers. (d) The graphs showing gene expression of core pluripotency markers in LCLs and their reprogrammed iPSCs. (e) PCR analysis of genomic DNA confirms no integration or retention of plasmid genome/transgene in the LCL reprogrammed iPSCs at passages 17–20. (f) Image showing immunocytochemistry analysis of the cells of three embryonic germ layers differentiated from reprogrammed iPSCs using monolayer differentiation protocol. (g) Image showing normal karyotype of an iPSC line. Karyotype analyses of each reprogrammed iPSC line were found to be normal. (h) The differential gene expression graph showing significant downregulation of LCL specific genes.

On day 0, that is, 24 hours before nucleofection, LCL cultures were split into 1 : 2 ratios to keep LCLs in log growth phase. On day 1, about one million cells were nucleofected with 2.5 *μ*g equal amount mixture of episomal plasmids (pCE-hOCT3/4, pCE-hSK, pCE-hUL, and pCE-mp53DD) encoding reprogramming factors (i.e., OCT3/4, SOX2, KLF4, L-MYC, and LIN28) and mouse p53DD (p53 carboxy-terminal dominant-negative fragment) as described by Okita et al. [24]. The episomal plasmids were obtained from Addgene nonprofit plasmid repository and nucleofection was performed using SE Cell Line 4D-Nucleofector® X Kit and 4D-Nucleofector DN-100 program on a 4D-Nucleofector system (Lonza, http://www.lonza.com/). The nucleofected LCLs were allowed to recover for 8–12 hours in 3 mL of RPMI 1640 complete media in a CO_2_ incubator at 37°C, 5% CO_2_, and atmospheric O_2_ and then transferred equally (i.e., 0.5 mL of media containing ~1.67 × 10^5^ cells) into a Matrigel® matrix (Corning Inc.) coated six-well plate containing 1 mL of TeSR*™*-E7 xeno-free reprogramming media (STEMCELL*™* Technologies). On days 3 and 5, 0.5 mL of fresh TeSR*™*-E7 media was added to each well and, on days 7 and 9, 1 mL of media was replaced with fresh TeSR*™*-E7 media. All precautions were taken as to not discard cells during media exchange. By days 7–9, cells start to adhere to the well surface and changes in cellular morphology were observed (Figure 1(a)). On day 11, spent media were replaced with 2 mL of fresh TeSR*™*-E7 media. Between days 13 and 15, when iPSC-like colonies started to appear, the cultures were transitioned to mTeSR*™*-1 (STEMCELL Technologies) hPSC maintenance media (Figure 1(a)). Media were changed daily thereafter. On days 18–21, 10–15 colonies morphologically similar to human ESCs (Figure 1(b)) and expressing surface antigen TRA-1-81 (live staining with TRA-1-81 antibody) were manually picked for further cultivation and evaluation.

Using this newly developed protocol, we achieved a consistently high reprogramming efficiency compared with the previously published LCL-to-iPSC reprogramming protocols (~50–200 colonies/million nucleofected cells), which enabled us to downsize our reprogramming experiments to approximately a third of the original (i.e., reducing the number of cells nucleofected and culture wells from ~1 million cells and 6-well format to only ~0.3 million cells and 2-culture-well format), thereby reducing the reprogramming media and other culture costs considerably. This constitutes a significant step towards the use of this technology in modeling human disease at a population scale.

The reprogrammed iPSC lines were confirmed by immunocytochemistry (Figure 1(c)) and differential gene expression analysis of the pluripotency markers using RNA-seq data (Figure 1(d)). The genomic/plasmid DNA PCR analysis was performed to confirm that the reprogrammed iPSCs were free from episomal plasmids used in LCL-to-iPSC reprogramming (Figure 1(e)). The functional competence of reprogrammed iPSC lines to differentiate into the cells of all three germ layers was assessed through monolayer differentiation into cells of endoderm, ectoderm, and mesoderm (Figure 1(f)) using the hPSC Functional Identification Kit (R&D Systems) and provided protocol with minor modifications. To assess the genomic integrity of the reprogrammed iPSCs, karyotyping (Figure 1(g)) was performed using the method described by Howe et al. [25] with minor modifications.

### 2.3. Genomic/Episomal Plasmid DNA PCR

Total cellular and plasmid DNA from snap-frozen cell pellets (~5 × 10^6^ cells) of the six stable reprogrammed iPSC lines was isolated using the DNeasy Blood and Tissue kit (Qiagen) according to the manufacturer's instructions. PCR to detect episomal plasmid DNA was performed for 30 cycles using primer pairs 5′-GGCGTAATCATGGTCATAGC-3′ and 5′-ACGACAGGTTTCCCGACT-3′ and Maxima Hot Start master mix (Thermo Scientific) following the manufacturer's instructions. The PCR primers were designed to amplify a genomic region common in all episomal plasmids used in our LCL-to-iPSC reprogramming method but not complimentary to human genome. Purified plasmid DNA was used as positive control. A genomic DNA fragment from a human single copy gene (*ALB*) was also PCR amplified using primer pairs 5′-TGAAACTTCGGCTCACTCCT-3′ and 5′-ATTGATGAAAAGGCGGTTG-3′ and following the same PCR conditions for normalization and as a positive control for cellular genome. The PCR products ware analyzed using 1% agarose gel electrophoresis.

### 2.4. Total RNA Extraction

RNA was extracted from cell pellets (~5 × 10^6^ cells) snap-frozen from LCLs immediately before nucleofection with reprogramming factors and from their stable reprogrammed iPSC lines. Total RNA was extracted from aforesaid LCL and iPSC lines (six each) using TRIzol reagent (Life Technologies) and the manufacturer protocol with minor modifications. RNA quality and quantity were assessed using a NanoDrop 2000 Spectrophotometer (Thermo Scientific) and an Agilent 2100 Bioanalyzer (Agilent Technologies).

### 2.5. RNA Sequencing and Data Preprocessing

#### 2.5.1. Small RNA Sequencing

The Illumina® TruSeq® Small RNA Sample Preparation Kit was used to prepare small RNA sequencing libraries from 1 *μ*g of total RNA. Briefly, the kit uses a modified RNA 3′ adaptor that specifically targets miRNA and other small RNAs that have a 3′ hydroxyl group. After ligation to each end, reverse transcription was performed to synthesize complementary single stranded cDNA. The cDNA library was then PCR amplified and gel purified for fragment size of 145–160 nucleotides containing miRNA and other regulatory small RNAs. The small RNA libraries from all the 12 samples (i.e., six LCLs and their reprogrammed iPSCs) were then deep sequenced using the Illumina HiSeq 2500 platform to generate 13.8 million sequence reads.

#### 2.5.2. mRNA Sequencing

The Illumina TruSeq RNA Sample Preparation Kit v2 was used to prepare cDNA sequencing libraries from 1 *μ*g of total RNA. Briefly, poly-A tail containing mRNA molecules was enriched from total RNA using oligo-dT attached magnetic beads. The mRNA enriched samples were fragmented into smaller pieces (~200–600 base pairs) using divalent cations under elevated temperature. The cleaved RNA fragments were then used as a template to synthesize first-strand cDNA using reverse transcriptase and random primers, followed by second-strand cDNA synthesis, using DNA polymerase-I and RNase H. The synthesized cDNA fragments were end-repaired and adaptor ligations were performed. The products were purified and cDNA libraries enriched with PCR were deep sequenced on the Illumina HiSeq 2500 platform to generate 58.3 million paired-end sequence reads across the 12 samples.

#### 2.5.3. Sequence Analysis

Raw fastq sequence files were generated and demultiplexed using the Illumina CASAVA v1.8 pipeline. After prealignment QCs, sequences were aligned to human genome build 19 (hg19) and mapped to UCSC transcripts using Strand NGS software v2.1 (Strand Genomics Inc.) with default settings. The small RNA reads were also mapped to small RNA annotations as implemented in Strand NGS v2.1 (Strand Genomics Inc.) The aligned reads were then filtered based on read quality metrics (i.e., quality threshold ≥ 20; N's allowed in read ≤ 1; mapping quality threshold ≥ 40; read length ≥ 20), so that only good alignments were retained and then quantification was performed. The expression values (read counts) were log transformed and “DESeq” normalization was applied. Only known mRNAs and miRNAs having the normalized read count (NRC) ≥ 20 in all samples of any one out of two cell types or in both (i.e., LCLs or iPSCs or both) were selected for differential expression analysis.

### 2.6. Differential Gene Expression Analysis

For both miRNA-seq and mRNA-seq datasets, we performed moderated *t*-statistics and expression fold change analysis using Strand NGS software. Based on the criteria fold change-absolute (FC-abs) ≥ 2.0 and false discovery rate adjusted *p* value (FDR) ≤ 0.05, differentially expressed genes were identified between LCLs and iPSCs.

### 2.7. Functional Annotations and Pathway Analysis

To identify biological functions that were most significant to our dataset, functional annotation enrichment analysis of significantly DE mRNAs and miRNAs was performed using IPA. Right-tailed Fisher's exact test *p* values corrected for FDR were used to calculate enrichment significance. The direction of functional change during LCL-to-iPSC reprogramming was assessed by activation* Z*-score as implemented in IPA. This allows the assessment of changes in respective cell type-specific key canonical pathways as a consequence of transcriptomic changes during LCL-to-iPSC reprogramming. The datasets comprising the normalized read count of the expressed genes (NRC ≥ 20) in LCLs and iPSCs and fold change values of significantly DE genes and miRNAs during iPSC reprogramming were analyzed in IPA and the predicted changes in the pathways were assessed by comparing the generated expressed and DE gene pathway visualization.

## 3. Results and Discussion

### 3.1. Human LCL-to-iPSC Reprogramming and Validation

Given the potential utility of the extensive LCL bioresource, we attempted reprogramming six LCLs from our SAFHS Mexican American participants into iPSCs using two previously published methods that demonstrated successful reprogramming of LCLs [15, 16]. In the first set of reprogramming experiments, we followed the method published by Choi et al. [15] and successfully nucleofected (assessed by parallel control nucleofection of GFP plasmid) one million cells per cell line with a 10 *μ*g mixture of EBNA1/OriP plasmid (i.e., 3.5 *μ*g of EN2L, 2.5 *μ*g of ET2K, and 4.0 *μ*g of EM2K) obtained from Addgene plasmid repository, encoding the OCT4, SOX2, NANOG, LIN28, c-Myc, KLF4, and SV40LT reprogramming factors. The nucleofected cells were cultured using the media and culture conditions described in the publication but after repeating the experiments twice on six different cell lines we did not achieve any success with this protocol. In the second set of experiments, we followed the method described by Rajesh et al. [16] and nucleofected the same six LCLs with a 2 *μ*g mixture of the different plasmid combination described in the publication. We achieved some success with OSNK, OSTK, and L-mL combinations. Only two out of six LCLs would be reprogrammed into iPSC using this method, but efficiency remained very poor (Table 1). Whilst working on these reprogramming experiments and trying different plasmid combinations, we have optimized an efficient LCL reprogramming protocol (see Figure 1(a) and the Materials and Methods for the detailed protocol), achieving 100% reprogramming success (our 29 reprogrammed iPSC lines) and very high reprogramming efficiency per nucleofection of one million cells (Table 1). We used equal amount mixture of episomal plasmids (pCE-hOCT3/4, pCE-hSK, pCE-hUL, and pCE-mp53DD) encoding reprogramming factors OCT3/4, SOX2, KLF4, L-MYC, LIN28, and mouse p53DD (p53 carboxy-terminal dominant-negative fragment). This combination constitutes two major changes from the previously published methods of Choi et al. [15] and Rajesh et al. [16]. The first change is the use of a mouse p53DD (p53 carboxy-terminal dominant-negative fragment) for* TP53* suppression.* TP53* is an important cell cycle regulator and it has been shown that its suppression enhances the iPSC reprogramming process both in fibroblasts [24] and in LCLs [23]. The second change we made is that we removed* SV40LT* from our reprogramming mix, which was used in all three previously published LCL-to-iPSC reprogramming methods by Choi et al. [15], Rajesh et al. [16], and Barrett et al. [23]. The expression of* SV40LT* was shown to be involved in suppression of iPSC induction [24]. Our optimized method achieves consistently higher LCL-to-iPSC reprogramming efficiency than the previously published methods. Furthermore, we used commercially available reprogramming media; therefore, our method can be easily reproduced by different laboratories interested in LCL-to-iPSC reprogramming. All of our six iPSC lines used in this study and 23 others were reprogrammed using our optimized method. The reprogrammed iPSC lines formed flat and compacted colonies and showed high nucleus-to-cytoplasm ratios, the typical morphology of human ESCs (Figure 1(b)). The immunocytochemistry and differential gene expression analysis showed that all of our reprogrammed iPSC lines express pluripotency markers (Figures 1(c) and 1(d)). The genomic/plasmid DNA PCR analysis showed that the reprogrammed iPSCs were free from episomal plasmids used in LCL-to-iPSC reprogramming at 17–20 passages (Figure 1(e)). The iPSCs also showed potential to differentiate into cells of all three germ layers by* in vitro* monolayer differentiation protocols and exhibited a normal karyotype (Figures 1(f) and 1(g)).

LCLs show high expression of the B-cell activation markers (FCER2/CD23, CD70, TNFRSF8/CD30, and ENTPD1/CD39) and cellular adhesion molecules (ITGAL/CD11a, LFA3/CD58, and ICAM1/CD54) [26]. These markers are usually absent or expressed at very low levels in resting B-cells, but their expression is significantly upregulated by EBV encoded nuclear antigens (EBNA2, EBNA3C) and latent membrane proteins (LMP1, LMP2A) when EBV infection is used to generate the LCLs [27–29]. The differential gene expression analysis of LCLs and their reprogrammed iPSCs shows significant downregulation of these markers in reprogrammed iPSCs (Figure 1(h)), which supports previous findings that EBV transcriptional activity is inhibited in the reprogrammed iPSCs [15, 16].

### 3.2. Differentially Expressed (DE) Genes

To investigate the mechanistic gene expression changes that occurred during LCL-to-iPSC reprogramming, we performed a parallel genome-wide miRNA and mRNA expression analysis in six LCLs and their reprogrammed iPSCs. A total of 5.5 and 8.3 million small RNA 40 bp single-end reads and 28.4 and 29.9 million mRNA 100 bp paired-end reads were obtained for LCLs and their reprogrammed iPSCs, respectively, from 24 cDNA libraries (12 each for small RNA and mRNA) sequenced on an Illumina HiSeq 2500 platform. Only known miRNA and mRNA genes/transcripts with NRC ≥ 20 (i.e., normalized value ≥ 4.3219 on log_2_ scale) in all samples of any one or both cell types (i.e., in LCLs or in iPSCs or in both) were considered to be expressed. We detected 12,325 mRNA and 116 miRNA expressed genes/transcripts in LCL and iPSC pairs during LCL-to-iPSC reprogramming.

**Figure 2:**
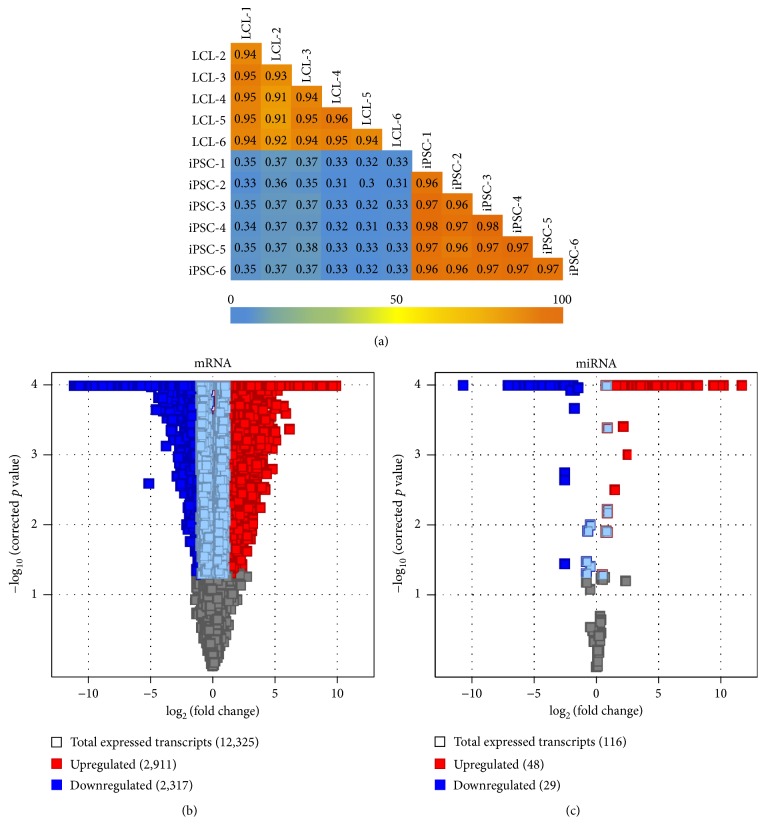
Differential gene expression statistics in LCL-to-iPSC reprogramming. (a) Expressed genes (NRC ≥ 20) correlation coefficient (*r* ^2^) plots for LCLs versus iPSCs. (b) Volcano plots showing differentially expressed genes (mRNAs) between LCLs and their reprogrammed iPSCs. (c) Volcano plots showing differentially expressed miRNAs between LCLs and their reprogrammed iPSCs.

Reproducibility of the expressed transcriptomic profiles in the biological replicates of each cell type was evaluated by calculating the correlation coefficient (*r* ^2^) on the total number of expressed genes/transcripts (Figure 2(a)) during LCL-to-iPSC reprogramming. The average correlation coefficient at 95% confidence interval (95% CI) was 0.938 ± 0.007 for LCL replicates and 0.969 ± 0.002 for iPSC replicates. These data suggest highly concordant resetting of gene expression profiles in all six cell lines/biological replicates during LCL-to-iPSC reprogramming.

To identify the unique transcriptomic signature of the LCL-to-iPSC reprogramming, we performed moderated *t*-statistics and expression fold change (FC) analysis in LCLs and their reprogrammed iPSCs. Using the FC-abs ≥ 2.0 and FDR ≤ 0.05 cutoffs, 5,228 mRNAs and 77 miRNAs were differentially expressed during LCL-to-iPSC reprogramming (Table S1 in Supplementary Material available online at http://dx.doi.org/10.1155/2016/2349261; Figures 2(b) and 2(c)). Among the mRNAs and miRNAs that were DE during iPSC reprogramming, 2,317 mRNAs and 29 miRNAs were downregulated and they accounted for the majority of the DE mRNAs and miRNAs reads (i.e., 85.6% and 97% reads, resp.) in LCLs. The upregulated 2,911 mRNAs and 48 miRNAs constituted most of the DE iPSC transcriptome reads (i.e., 80% mRNA and 96% miRNA reads).

**Figure 3:**
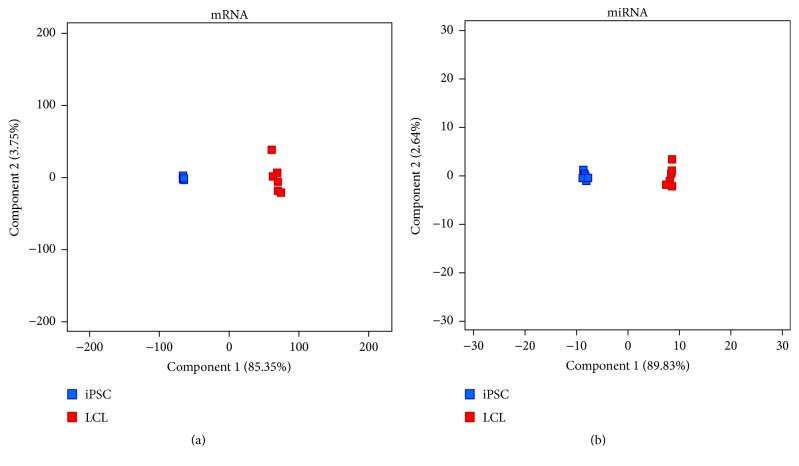
Principal component analysis (PCA) based on DE genes. (a) DE mRNAs during LCL-to-iPSC reprogramming. (b) DE miRNAs during LCL-to-iPSC reprogramming.

Principal component analysis (PCA) of differentially expressed mRNAs and miRNAs during LCL-to-iPSC reprogramming is shown in Figure 3. The first principal component (Component 1), which represents the expression variance due to reprogramming (i.e., expression change as a function of LCL and iPSC cellular identities), accounts for 85.35% of the variance observed in DE mRNAs (Figure 3(a)) and 89.83% of the variance observed in DE miRNAs (Figure 3(b)) during iPSC reprogramming.

These results suggest discrete and uniform resetting of both mRNA and miRNA expression during iPSC reprogramming, each cell type expressing a unique set of genes and miRNAs.

### 3.3. Transcriptomic and Functional Signature of LCL-to-iPSC Reprogramming

**Figure 4:**
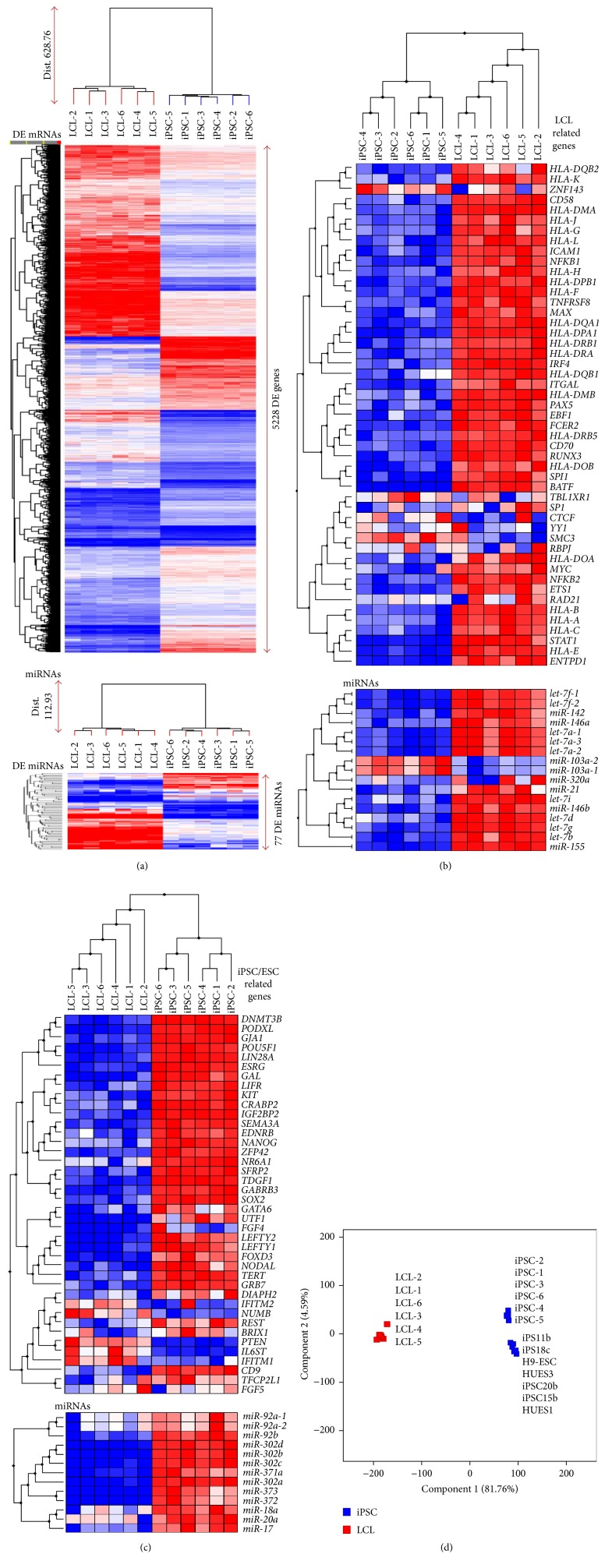
Gene expression patterns in LCLs, reprogrammed iPSC, and ESCs. (a) Expression pattern of all DE mRNAs and miRNAs in LCLs and their reprogrammed iPSCs. (b) Expression profiles of LCL specific mRNAs and miRNAs in LCLs and their reprogrammed iPSCs. (c) Expression profiles of mRNAs and miRNAs known from the literature to be involved in maintenance of pluripotency and stemness in human ESC/iPSCs. (d) PCA on gene expression profiles of six LCLs and their reprogrammed iPSCs generated in this study and three ESC and four iPSC gene expression profiles downloaded from the GEO database.

Hierarchical clustering based on the expression profiles of significantly DE genes and miRNAs during LCL-to-iPSC reprogramming is shown in Figure 4(a).

#### 3.3.1. LCL Related Genes

During* in vitro* EBV immortalization of B-lymphocytes, EBV oncoprotein expression converts resting B-lymphocytes into LCLs, which consequently show higher expression of B-cell activation markers, similar to antigenic or mitogenic stimulation of the resting B-cells [26, 28]. The LCL ChIP-seq data also indicate that EBV evolved to usurp B-cell-intrinsic activation programs to support rapid growth and survival of latently infected B-cells/LCLs [30]. Therefore, we explored the fate of EBV latent oncoprotein's transcriptomic effect [29, 31] in our reprogrammed iPSCs which includes the known effects on B-cell transcription factors (*STAT1, MYC, YY1, SP1, PAX5, BATF, IRF4, ETS1, RAD21, SPI1, CTCF, RBPJ, ZNF143, SMC3, NFKB1, NFKB2, TBL1XR1, EBF1, MAX,* and* RUNX3*), major histocompatibility complex classes I and II, cell surface markers (*FCER2/CD23, CD70, TNFRSF8/CD30,* and* ENTPD1/CD39*), cellular adhesion molecules (*ITGAL/CD11a, LFA3/CD58,* and* ICAM1/CD54*), and LCL specific miRNAs (*miR-155, let-7a-i, miR-21, miR-142, miR-103, miR-320,* and* miR-146a-b*). These B-cell/LCL specific markers (mRNA and miRNA) were highly enriched in differentially expressed genes and showed significant downregulation in reprogrammed iPSCs (Figure 4(b)). A few of the B-cell transcription factors (*YY1, SP1, RAD21, CTCF, RBPJ, ZNF143, SMC3,* and* TBL1XR1*) that were highly expressed in both LCLs and their reprogrammed iPSCs may perform vital cellular functions; namely,* YY1* and* SP1* are ubiquitously expressed transcription factors that are involved in repressing and activating a diverse number of promoters [32, 33]; cohesin component* RAD21* as well as* SMC3* exhibits a functional role in maintaining ESC identity through association with the pluripotency transcriptional network [34];* CTCF* is involved in many cellular processes, including transcriptional regulation, insulator activity, and regulation of chromatin architecture [35];* RBPJ* DNA binding protein plays a role in lineage specification and stem cell expansion [36];* ZNF143* is an important regulator of mammalian embryonic stem cell renewal [20];* miR-103a* is expressed in various cell lineages and may perform some basic cellular functions [37].

#### 3.3.2. iPSC/ESC Related Genes

The genes and miRNAs expected to be enriched in iPSCs/ESCs, from the literature [18, 21, 38–42], include transcription factors involved in maintaining “stemness” (*FOXD3, GATA6, NANOG, NR6A1, POU5F1, SOX2, UTF1, TFCP2L1,* and* ZFP42*), signaling molecules involved in pluripotency and self-renewal (*CRABP2, EDNRB, FGF4, FGF5, GABRB3, GAL, GRB7, IFITM1, IL6ST, KIT, LEFTY1, LEFTY2, LIFR, NODAL, NOG, NUMB, PTEN, SFRP2,* and* TDGF1*), cytokines and growth factors (*FGF4, FGF5, LEFTY1, LEFTY2, NODAL,* and* TDGF1*), other ESC-specific genes (*BRIX1, CD9, DIAPH2, DNMT3B, IFITM2, IGF2BP2, LIN28A, PODXL, REST, SEMA3A, TERT, ESRG,* and* GJA1*), and miRNAs (*miR-302a, miR-302c, miR-371a, miR-302b, miR-302d, miR-372, miR-373, miR-92a-1, miR-92a-2, miR-92b, miR-17, miR-20a,* and* miR-18a*) that were highly enriched in genes and miRNAs that were expressed (NRC ≥ 20) in our reprogrammed iPSCs and the majority of them showed significant upregulation (FC ≥ 2.0, FDR ≤ 0.05) during iPSC reprogramming (Figure 4(c)). The expression of transcription factors* GATA6* and* UTF1,* signaling molecule* NODAL*, and growth factor* FGF4* showed upregulation during iPSC reprogramming; however, it was below the detection threshold (NRC ≤ 20) in one or more of our iPSCs, possibly due to lower overall read counts. The expression of a few iPSC/ESC markers (gene/miRNAs) either did not change significantly (*IFITM2, IFITM1, DIAPH2, NUMB, REST, BRIX1, TFCP2L1, FGF5*, and miR-92a) or was significantly (FC ≤ −2.0, FDR ≤ 0.05) downregulated (*PTEN* and* IL6ST*) in our reprogrammed iPSCs.* IFITM1* and* IFITM2* are interferon-induced antiviral cell-intrinsic restriction factors with high constitutive expression in many cells. Both* IFITM1* and* IFITM2* were highly expressed (NRC > 100) in both our LCLs and reprogrammed iPSCs. EBV oncoprotein in LCLs and* OCT4/POU5F1* in iPSCs synergistically facilitate the expression of endogenous retroviral integration elements in the human genome, which consequently increase expression of* IFITM* proteins in both LCLs and iPSCs [43, 44]. The* DIAPH2 gene* is ubiquitously expressed and affects cytokinesis and other actin-mediated morphogenetic processes that are required in self-renewal and early steps of development [45]. The* NUMB* gene's primary function in cell differentiation is as an inhibitor of Notch signaling which is essential for maintaining self-renewal potential in stem and progenitor cells [46], whereas it acts as a coactivator of EBNA2 activity by suppressing Notch signaling in LCLs [47]. The* REST* gene encodes a transcriptional repressor of neuronal genes in nonneuronal tissues and was expressed in our LCLs and iPSCs. However, its role in self-renewal and pluripotency of ESCs remains ambiguous [48–50]. The* TFCP2L1* is preferentially expressed in ESCs and upregulates* NANOG* expression and promotes self-renewal in a* NANOG* dependent manner [51, 52].* miR-92a1* and* miR-92a2* were among the most abundant miRNAs in our LCLs and reprogrammed iPSCs.* miR-92a* are ubiquitously expressed in majority of cell types and target genes involved in cell cycle regulation and cell signaling and thus are necessary during all stages of mammalian development and essential for the proliferation of cells [53]. The* PTEN* gene was highly expressed (NRC > 100) both in LCLs and in reprogrammed iPSCs but its expression was downregulated during the LCL-to-iPSC reprogramming.* PTEN* is a key regulator of hESC growth and differentiation [54]. The pathways of* CXCR4* and* PTEN* converge, leading to the promotion and regulation of tumorigenesis [55], and* CXCR4* expression is inhibited by EBV oncoproteins in LCLs. Based on these observations, we hypothesized that* PTEN* is highly upregulated by EBV oncoproteins in LCLs. Like* PTEN*,* IL6ST* was also highly expressed both in LCLs and in iPSCs but was downregulated during iPSC reprogramming. EBV oncoprotein LMP1 is known to significantly upregulate* IL6ST* in LCLs [56]. In iPSCs/ESCs,* IL6ST* is primarily associated with cell survival and differentiation [57].

#### 3.3.3. Principal Component Analysis of Gene Expression Profiles between LCLs, Their Reprogrammed iPSC Lines, and Other ESC and iPSC Lines Available from Public Domain

To further assess and compare the gene expression profile of our LCL reprogrammed iPSCs with other ESC and iPSC line gene expression profiles, we downloaded whole genome RNA sequencing data of three ESC lines, that is, GSM1888661 (H9ESC), GSM1888664 (HUES1), and GSM1888680 (HUES3), and four iPSC lines, that is, GSM1888662 (iPS11b), GSM1888660 (iPS15b), GSM1888679 (iPS18c), and GSM1888663 (iPS20b), from GEO public database submitted by Choi et al. [58]. Principal component analysis of the expressed mRNAs of 6 LCLs, their reprogrammed iPSCs (this study), and 3 ESCs and 4 iPSCs (downloaded from GEO database) is shown in Figure 4(d). The first principal component (Component 1) which represents the expression variance between LCLs and ESCs/iPSCs (6 iPSCs (this study) and 3 ESC and 4 iPSC expression profiles downloaded from GEO database) clusters accounts for 81.76% of the total variance observed in all expressed mRNAs. The second principal component (Component 2), which represents the expression variance between the ESC/iPSC downloaded from GEO database and the LCL reprogrammed iPSC lines generated in this study, accounts for only 4.59% of the variance observed in all expressed mRNAs. This small variance in the expressed mRNAs of our reprogrammed iPSCs and ESCs/iPSCs data downloaded from GEO may be attributed to the differences between the genetic backgrounds of the donors of these lines as well as laboratory-to-laboratory variation. This analysis further confirms that our LCL reprogrammed iPSCs have a very similar gene expression profile to that of human ESCs and iPSCs.

#### 3.3.4. Functional Annotation of DE Genes and miRNAs

**Figure 5:**
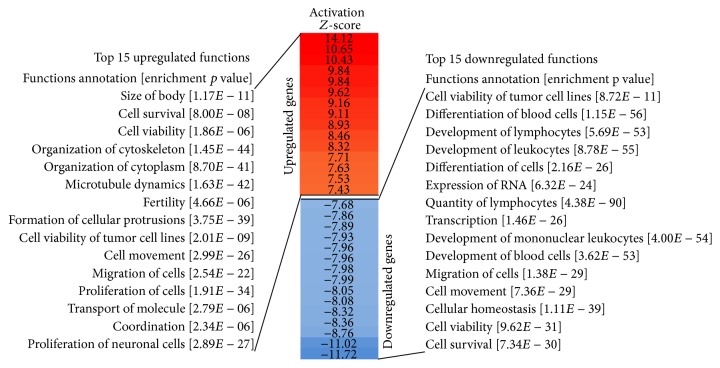
Graphical presentation of the top 15 upregulated and downregulated cellular functions found to be enriched during LCL-to-iPSC reprogramming.

To better understand what biological functions were affected and how these were affected by differentially expressed mRNAs and miRNAs during LCL-to-iPSC reprogramming, we performed functional annotation enrichment analysis of downregulated and upregulated mRNAs and miRNAs using IPA. The significantly enriched (FDR ≤ 0.001) functions that were also either significantly upregulated (activation* Z*-score ≥ 2.0) or downregulated (activation* Z*-score ≤ −2.0) consequent to the upregulation or downregulation of the DE genes and DE miRNAs are presented in Supplementary Figure S1 and Supplementary Table S2. The top 15 upregulated and downregulated functions are presented in Figure 5. The 224 biological functions that were significantly enriched in downregulated genes and miRNAs predominantly showed deactivation/downregulation of hematologic system development and functions, particularly the functions related to lymphocytes. This suggests that during reprogramming LCLs lose their B-lymphocyte identity/functions. The 161 biological functions that were enriched in the upregulated DE genes and miRNAs predominantly showed the activation/upregulation of early organism development suggesting that reprogrammed iPSCs possess functional profiles very similar to ESCs. Some of the basic cellular functions (e.g., cell proliferation, survival, viability, movement/migration, phosphorylation of proteins, and organismal death) were enriched in both upregulated and downregulated genes and miRNAs but contrarily regulated. Both LCL and reprogrammed iPSC share a basic self-renewal property; however, these results suggest that such shared property may be regulated very differently in LCLs and their reprogrammed iPSCs.

#### 3.3.5. Canonical Pathways in LCL-to-iPSC Reprogramming

Further, we explored the effects of iPSC reprogramming on key LCL and human iPSC related canonical pathways using IPA platform.

Previous studies of the molecular genetics and pathogenesis of EBV induced B-cell growth support a model where EBV encoded nuclear antigens (EBNA1, EBNA2, and EBNA3A-C) and integral membrane proteins (LMP1 and LMP2) utilize intrinsic B-cell receptors (BCR) signaling pathways to support rapid growth and survival of latently infected B-cells/LCLs [26, 28, 30, 59].

**Figure 6:**
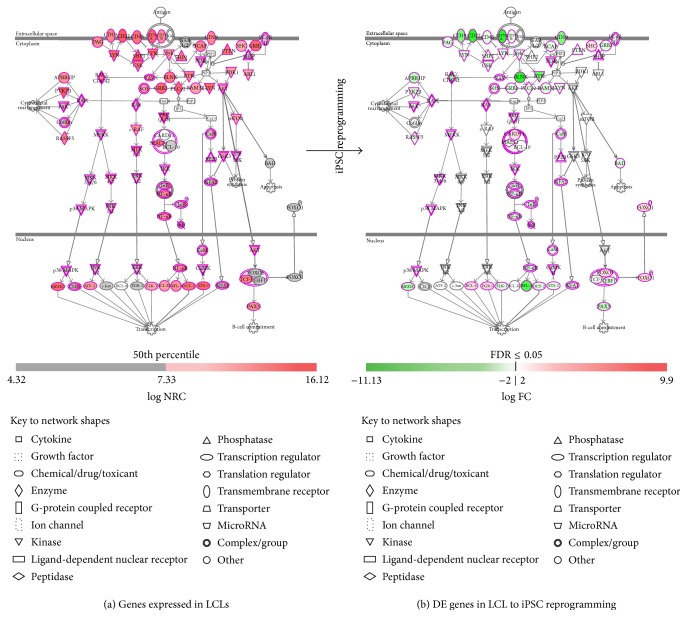
Diagram showing key LCL specific canonical pathways. (a) Pathway genes expressed in LCLs. (b) Differential expression of pathway genes during LCL-to-iPSC reprogramming.

The EBV principal oncoprotein LMP1 along with LMP2A mimics CD40 and B-cell receptor (BCR) signaling, respectively, and activates NF-*κ*B, JNK, and MAPK pathways [29, 60–63]. These pathways control B-lymphocyte proliferation, differentiation, and survival and are critically important regulators of normal and pathological innate and adaptive immune responses mediated through BCR signaling. As expected, almost all of these pathways were in an activated state in our LCLs (Figure 6(a)) and were significantly downregulated in reprogrammed iPSCs (Figure 6(b)). The FOXO1 and MEK/ERK signaling which was upregulated both in LCLs and in reprogrammed iPSCs is known to play a role in human iPSC/ESC maintenance [64, 65].

**Figure 7:**
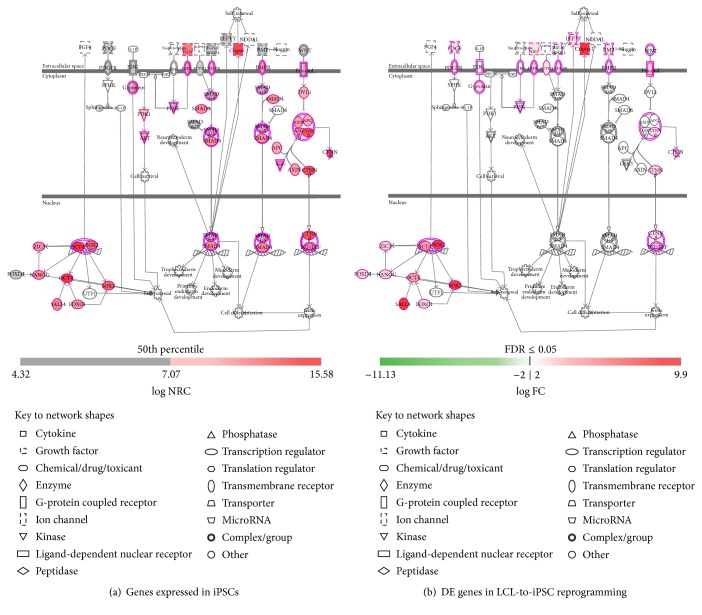
Diagram showing key human pluripotency pathways in ESCs/iPSCs. (a) Pathway genes expressed in reprogrammed iPSCs. (b) Differential expression of pathway genes during LCL-to-iPSC reprogramming.

Human iPSCs have similar properties to human ESCs (hESCs), such as self-renewal and differentiation capacity [13, 66]; therefore, we explored the state of human ESC pluripotency pathways in our reprogrammed iPSCs. Human iPSCs/ESCs exhibit a number of signaling pathways involved in self-renewal and pluripotency, regulated by a combination of intrinsic and extrinsic factors. Intrinsic factors include a group of core transcription factors, that is, POU5F1/OCT4, SOX2, and NANOG. The OCT3/4 and SOX2 partnership is indispensable in the maintenance of pluripotency [67, 68]. NANOG is another member of the group of transcription factors whose functions are deemed essential for the process of self-renewal in human ESCs [69, 70]. All the core pluripotency transcription factor's genes (i.e.,* POU5F1/OCT4, SOX2*, and* NANOG*) were highly expressed (Figure 7(a)) and significantly upregulated (Figure 7(b)) in our reprogrammed iPSCs. The extrinsic basic FGF (bFGF) allows the clonal growth of human ESCs/iPSCs on fibroblasts in the presence of commercially available serum replacement [71]. At higher concentrations, bFGF allows feeder independent growth of human ESCs/iPSCs cultured in the same serum replacement [72–75]. Apart from the core transcription factors, FGF signaling and a balance between TGF-*β*/Activin and BMP signaling are central to the self-renewal of human ESCs/iPSCs [76–78]. The TGF-*β* superfamily of ligands plays a major role in maintaining the self-renewing capacity of human ESCs/iPSCs through two main branches: the SMAD1/5 branch which is transduced on behalf of BMP and GDF ligands via type I receptors ALK1, ALK2, ALK3, and ALK6 and the TGF-*β*/Activin/NODAL branch, which involves the activation of SMAD2/3 via ALK4, ALK5, and ALK7 [79–83]. There are also two inhibitory SMADs: SMAD6, which selectively inhibits SMAD1/5, and SMAD7, which inhibits TGF-*β* signaling [84–86]. Upon activation by phosphorylation and association with a common SMAD4, receptor activated SMADs translocate to the nucleus and in concert with other transcription factors regulate gene expression. SMAD2/3 pathway is also required for positive regulation of several factors of TGF-*β* signaling. These factors include NODAL, CRIPTO, LEFTY1, and LEFTY2 [83]. However, genes involved both in TGF-*β*/Activin and in BMP signaling pathways were highly expressed and the expression of TGF-*β*-responsive genes* NODAL, CRIPTO*, and* LEFTY* was also significantly upregulated in our reprogrammed iPSCs (Figure 7(b)). The expression of TGF-*β* itself was downregulated and the expression of most SMADs did not change significantly during LCL-to-iPSC reprogramming (Figure 7(b)). In LCLs, EBV encoded EBNA1 represses TGF-*β* induced gene transcription through rapid degradation of SMAD2 protein; however, it does not affect the other SMAD proteins or the transcription of either TGF-*β* or SAMD2 itself [87]. In normal cells, activated SMAD complex consisting of either dimers or trimers of phosphorylated SMAD2/3 bound to SMAD4 is responsible for subsequent transcriptional regulation of TGF-*β*-responsive genes [88]. The TGF-*β*-responsive genes function in both autocrine and paracrine manners and control the expression of their upstream regulator TGF-*β* and SMADs [89]. In the absence of TGF-*β*-responsive proteins, transcription of TGF-*β* and SMADs (particularly* SMAD2, SMAD3*, and* SMAD4*) was highly upregulated in LCLs, whereas, in reprogrammed iPSCs, EBNA1 transcription was inhibited and therefore TGF-*β* induced gene transcription was reinstated (as shown by upregulated* NODAL, CRIPTO*, and* LEFTY*), which along with extrinsic TGF-*β* in the iPSC maintenance media plausibly downregulated TGF-*β* transcription slightly, but mRNA levels of most SMADs did not change significantly indicating that TGF-*β* signaling plays an important role in maintenance of human ESC/iPSCs.

In contrast to TGF-*β*/Activin/NODAL signaling, high BMP activity is associated with differentiation of human ESCs/iPSCs. Repression of BMP signaling in human ESCs/iPSCs by Noggin and FGF supports long-term self-renewal [74]. Binding of FGF to its receptor and heparin leads to receptor autophosphorylation and activation of intracellular signaling cascades, including the Ras/ERK pathway and the PI3K pathway [64, 90, 91]. Our reprogrammed iPSCs show evidence of FGF induced activation of both Ras/ERK (Figure 6(b)) and PI3K (Figure 7(a)) pathways.

Wnt/*β*-catenin signaling also plays an important role in controlling ESC maintenance. Canonical Wnt signaling involves the binding of Wnt to the frizzled receptors. This, in turn, activates Dsh, which displaces GSK3*β* from the APC/AXIN complex, preventing ubiquitin mediated degradation of *β*-catenin. Subsequently, *β*-catenin accumulates and translocates to the nucleus where it associates with TCF/LEF to activate transcription of Wnt target genes [92, 93]. All genes in Wnt/*β*-catenin signaling pathway were highly expressed in our reprogrammed iPSCs and expression of *β*-catenin was significantly upregulated (Figures 7(a) and 7(b)).

Our reprogrammed iPSCs also showed evidence of activation of the S1P signaling pathway (Figure 7(b)). S1P signals both extracellularly through EDG receptors coupled to G-proteins and intracellularly by unidentified mechanisms that support human ESC/iPSC self-renewal [94].

#### 3.3.6. Recovery of Donor's Genetic Relationships and Disease State

**Figure 8:**
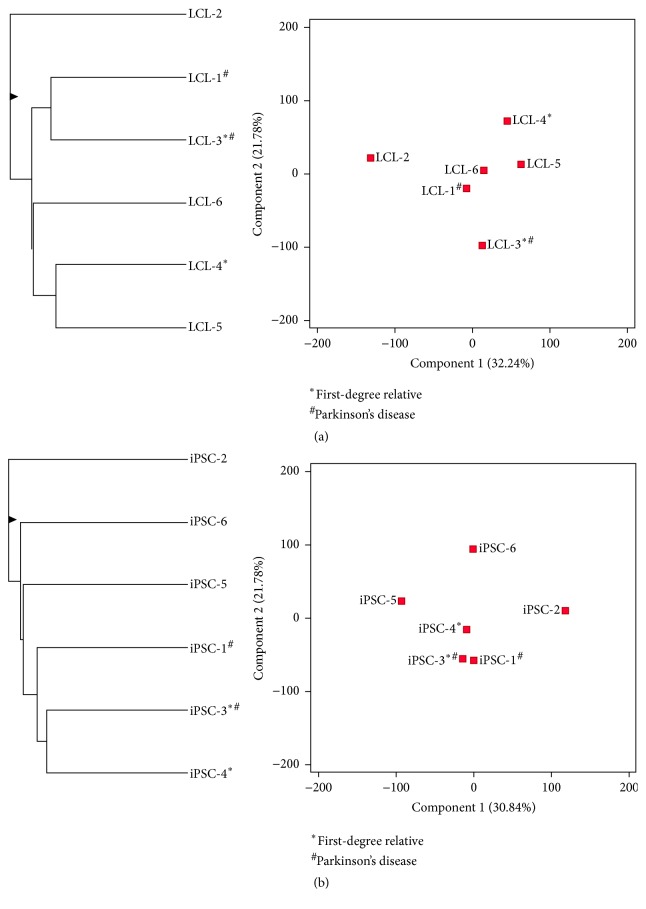
Clustering properties of LCLs and their reprogrammed iPSCs (a) Hierarchical clustering analysis and PCA in LCLs based on all genes detected as expressed during LCL-to-iPSC reprogramming. (b) Hierarchical clustering analysis and PCA in generated iPSCs based on all genes detected as expressed during LCL-to-iPSC reprogramming. ^#^The donors of LCL-1 and LCL-3 were diagnosed with sporadic Parkinson's disease. The donors of LCL-2, LCL-4, LCL-5, and LCL-6 were healthy. ^*∗*^The donors of LCL-3 and LCL-4 were first-degree relatives whereas all other donors were unrelated.

Because the regulation of a large number of genes was affected by iPSC reprogramming (42.4% of the total expressed genes), we investigated whether the gene expression pattern specific to the donor's genetic relationships and disease state was recovered in the process. We performed hierarchical clustering analysis and PCA using data on all 12,325 mRNAs detected as expressed in LCLs and their reprogrammed iPSCs. The LCLs fail to consistently cluster by the genetic relationships of their donors or by the disease state (Figure 8(a)). The similarity of iPSC-3 and iPSC-4 is driven by genetic relatedness since these donors are first-degree relatives sharing 50% of their genetic background (Figure 8(b)). The similarity of iPSC-1 and iPSC-3, which are unrelated donors with Parkinson's disease, exhibits the second most minimal distance from each other (hierarchical clustering, Figure 8(b)). This proximity appears to be driven by shared disease state. Further, we performed differential gene expression analysis between iPSC lines of Parkinson's patients and iPSC lines of healthy donors. Using the FC-abs ≥ 2.0 and FDR ≤ 0.05 cutoffs, no genes were found to be differentially expressed. More relevant neuronal cells will be differentiated from the generated iPSCs, to further investigate the pathophysiology and genetics of these Parkinson's disease cases. Several previous studies have shown successful modeling of Parkinson's disease in neurons generated from iPSCs [95–104].

## 4. Conclusions

To enable the utilization of existing LCL bioresources in iPSC based disease modeling, it is an absolute necessity to develop an efficient and reproducible LCL-to-iPSC reprogramming method. Here, we describe a MEF feeder-free protocol for efficient and reproducible reprogramming of iPSCs from LCL using publically available plasmids and commercially available media. In addition, our comprehensive analysis of genome-wide miRNome and transcriptome of LCLs and their reprogrammed iPSCs provides important documentation of differentially expressed genes and miRNAs and their functional consequences during LCL-to-iPSC reprogramming which were previously unknown.

## Supplementary Material

Table S1: miRNA and mRNA genes that showed significant differential expression between LCLs and reprogrammed iPSCs.
Table S2: Functional annotation of the differentially expressed genes/miRNAs between LCLs and their reprogrammed iPSCs.
Figure S1: Graphical presentation of the cellular functions found enriched during LCL to iPSC reprogramming.

## Figures and Tables

**Table 1:** iPSC induction efficiency from six LCL lines.

Cell line	Cell number nucleofected	Reprogramming efficiency (%)/plasmid mixture and conditions		
		EN2L + ET2K + EM2K after Choi et al. [15]	OSNK + OSTK + L-mL after Rajesh et al. [16]	Our optimized protocol (see Materials and Methods)
LCL-1	1 × 10^6^	0.0	0.0003	0.0102
LCL-2	1 × 10^6^	0.0	0.0	0.0204
LCL-3	1 × 10^6^	0.0	0.0001	0.0054
LCL-4	1 × 10^6^	0.0	0.0	0.0108
LCL-5	1 × 10^6^	0.0	0.0	0.0132
LCL-6	1 × 10^6^	0.0	0.0	0.0216

## Additional Points

The datasets supporting the conclusion of this paper were submitted to GEO archive (gene expression omnibus) and a GEO accession number was assigned: GSE74289.

## References

[1] Hu V. W., Frank B. C., Heine S., Lee N. H., Quackenbush J. (2006). Gene expression profiling of lymphoblastoid cell lines from monozygotic twins discordant in severity of autism reveals differential regulation of neurologically relevant genes. *BMC Genomics*.

[2] Huang R. S., Duan S., Kistner E. O., Hartford C. M., Dolan M. E. (2008). Genetic variants associated with carboplatin-induced cytotoxicity in cell lines derived from Africans. *Molecular Cancer Therapeutics*.

[3] Moyer A. M., Fridley B. L., Jenkins G. D. (2011). Acetaminophen-NAPQI hepatotoxicity: a cell line model system genome-wide association study. *Toxicological Sciences*.

[4] Ziliak D., O'Donnell P. H., Im H. K. (2011). Germline polymorphisms discovered via a cell-based, genome-wide approach predict platinum response in head and neck cancers. *Translational Research*.

[5] Wen Y., Gamazon E. R., Bleibel W. K. (2012). An eQTL-based method identifies CTTN and ZMAT3 as pemetrexed susceptibility markers. *Human Molecular Genetics*.

[6] Pickrell J. K., Marioni J. C., Pai A. A. (2010). Understanding mechanisms underlying human gene expression variation with RNA sequencing. *Nature*.

[7] Banovich N. E., Lan X., McVicker G. (2014). Methylation QTLs are associated with coordinated changes in transcription factor binding, histone modifications, and gene expression levels. *PLoS Genetics*.

[8] NIMH Repository and Genomics Resource (2014). *NIMH-RGR Data Explorer*.

[9] Hannula K., Lipsanen-Nyman M., Scherer S. W., Holmberg C., Höglund P., Kere J. (2001). Maternal and paternal chromosomes 7 show differential methylation of many genes in lymphoblast DNA. *Genomics*.

[10] Carter K. L., Cahir-McFarland E., Kieff E. (2002). Epstein-Barr virus-induced changes in B-lymphocyte gene expression. *Journal of Virology*.

[11] Min J. L., Barrett A., Watts T. (2010). Variability of gene expression profiles in human blood and lymphoblastoid cell lines. *BMC Genomics*.

[12] Çalişkan M., Cusanovich D. A., Ober C., Gilad Y. (2011). The effects of EBV transformation on gene expression levels and methylation profiles. *Human Molecular Genetics*.

[13] Takahashi K., Tanabe K., Ohnuki M. (2007). Induction of pluripotent stem cells from adult human fibroblasts by defined factors. *Cell*.

[14] Yu J., Vodyanik M. A., Smuga-Otto K. (2007). Induced pluripotent stem cell lines derived from human somatic cells. *Science*.

[15] Choi S. M., Liu H., Chaudhari P. (2011). Reprogramming of EBV-immortalized B-lymphocyte cell lines into induced pluripotent stem cells. *Blood*.

[16] Rajesh D., Dickerson S. J., Yu J., Brown M. E., Thomson J. A., Seay N. J. (2011). Human lymphoblastoid B-cell lines reprogrammed to EBV-free induced pluripotent stem cells. *Blood*.

[17] Fong Y. W., Inouye C., Yamaguchi T., Cattoglio C., Grubisic I., Tjian R. (2011). A DNA repair complex functions as an Oct4/Sox2 coactivator in embryonic stem cells. *Cell*.

[18] Jaenisch R., Young R. (2008). Stem cells, the molecular circuitry of pluripotency and nuclear reprogramming. *Cell*.

[19] Boyer L. A., Tong I. L., Cole M. F. (2005). Core transcriptional regulatory circuitry in human embryonic stem cells. *Cell*.

[20] Chen X., Xu H., Yuan P. (2008). Integration of external signaling pathways with the core transcriptional network in embryonic stem cells. *Cell*.

[21] Kim J., Chu J., Shen X., Wang J., Orkin S. H. (2008). An extended transcriptional network for pluripotency of embryonic stem cells. *Cell*.

[22] Marson A., Levine S. S., Cole M. F. (2008). Connecting microRNA genes to the core transcriptional regulatory circuitry of embryonic stem cells. *Cell*.

[23] Barrett R., Ornelas L., Yeager N. (2014). Reliable generation of induced pluripotent stem cells from human lymphoblastoid cell lines. *Stem Cells Translational Medicine*.

[24] Okita K., Yamakawa T., Matsumura Y. (2013). An efficient nonviral method to generate integration-free human-induced pluripotent stem cells from cord blood and peripheral blood cells. *Stem Cells*.

[25] Howe B., Umrigar A., Tsien F. (2014). Chromosome preparation from cultured cells. *Journal of Visualized Experiments*.

[26] Rowe M., Rowe D. T., Gregory C. D. (1987). Differences in B cell growth phenotype reflect novel patterns of Epstein-Barr virus latent gene expression in Burkitt's lymphoma cells. *The EMBO Journal*.

[27] Wang F., Gregory C., Sample C. (1990). Epstein-Barr virus latent membrane protein (LMP1) and nuclear proteins 2 and 3C are effectors of phenotypic changes in B lymphocytes: EBNA-2 and LMP1 cooperatively induce CD23. *Journal of Virology*.

[28] Young L. S., Murray P. G. (2003). Epstein-Barr virus and oncogenesis: from latent genes to tumours. *Oncogene*.

[29] Kang M.-S., Kieff E. (2015). Epstein-Barr virus latent genes. *Experimental and Molecular Medicine*.

[30] Zhou H., Schmidt S. C. S., Jiang S. (2015). Epstein-barr virus oncoprotein super-enhancers control B cell growth. *Cell Host and Microbe*.

[31] Skalsky R. L., Corcoran D. L., Gottwein E. (2012). The viral and cellular microRNA targetome in lymphoblastoid cell lines. *PLoS Pathogens*.

[32] Tan N. Y., Khachigian L. M. (2009). Sp1 phosphorylation and its regulation of gene transcription. *Molecular and Cellular Biology*.

[33] Golebiowski F. M., Górecki A., Bonarek P., Rapala-Kozik M., Kozik A., Dziedzicka-Wasylewska M. (2012). An investigation of the affinities, specificity and kinetics involved in the interaction between the Yin Yang 1 transcription factor and DNA. *FEBS Journal*.

[34] Nitzsche A., Paszkowski-Rogacz M., Matarese F. (2011). RAD21 cooperates with pluripotency transcription factors in the maintenance of embryonic stem cell identity. *PLoS ONE*.

[35] Phillips J. E., Corces V. G. (2009). CTCF: master weaver of the genome. *Cell*.

[36] Meier-Stiegen F., Schwanbeck R., Bernoth K. (2010). Activated Notch1 target genes during embryonic cell differentiation depend on the cellular context and include lineage determinants and inhibitors. *PLoS ONE*.

[37] Suh M.-R., Lee Y., Kim J. Y. (2004). Human embryonic stem cells express a unique set of microRNAs. *Developmental Biology*.

[38] Cai J., Chen J., Liu Y. (2006). Assessing self-renewal and differentiation in human embryonic stem cell lines. *STEM CELLS*.

[39] Sharova L. V., Sharov A. A., Piao Y. (2007). Global gene expression profiling reveals similarities and differences among mouse pluripotent stem cells of different origins and strains. *Developmental Biology*.

[40] Efroni S., Duttagupta R., Cheng J. (2008). Global transcription in pluripotent embryonic stem cells. *Cell Stem Cell*.

[41] Young R. A. (2011). Control of the embryonic stem cell state. *Cell*.

[42] Zhao W., Ji X., Zhang F., Li L., Ma L. (2012). Embryonic stem cell markers. *Molecules*.

[43] Hsiao F. C., Lin M., Tai A., Chen G., Huber B. T. (2006). Cutting edge: epstein-Barr virus transactivates the HERV-K18 superantigen by docking to the human complement receptor 2 (CD21) on primary B cells. *Journal of Immunology*.

[44] Grow E. J., Flynn R. A., Chavez S. L. (2015). Intrinsic retroviral reactivation in human preimplantation embryos and pluripotent cells. *Nature*.

[45] Bione S., Sala C., Manzini C. (1998). A human homologue of the *Drosophila melanogaster* diaphanous gene is disrupted in a patient with premature ovarian failure: evidence for conserved function in oogenesis and implications for human sterility. *American Journal of Human Genetics*.

[46] Katoh M., Katoh M. (2006). NUMB is a break of WNT—notch signaling cycle. *International Journal of Molecular Medicine*.

[47] Gross H., Barth S., Pfuhl T. (2011). The NP9 protein encoded by the human endogenous retrovirus HERV-K(HML-2) negatively regulates gene activation of the Epstein-Barr virus nuclear antigen 2 (EBNA2). *International Journal of Cancer*.

[48] Singh S. K., Kagalwala M. N., Parker-Thornburg J., Adams H., Majumder S. (2008). REST maintains self-renewal and pluripotency of embryonic stem cells. *Nature*.

[49] Jørgensen H. F., Chen Z.-F., Merkenschlager M., Fisher A. G. (2009). Is REST required for ESC pluripotency?. *Nature*.

[50] Buckley N. J., Johnson R., Sun Y.-M., Stanton L. W. (2009). Is REST a regulator of pluripotency?. *Nature*.

[51] Ivanova N., Dobrin R., Lu R. (2006). Dissecting self-renewal in stem cells with RNA interference. *Nature*.

[52] Ye S., Li P., Tong C., Ying Q.-L. (2013). Embryonic stem cell self-renewal pathways converge on the transcription factor Tfcp2l1. *The EMBO Journal*.

[53] Uziel T., Karginov F. V., Xie S. (2009). The miR-17∼92 cluster collaborates with the Sonic Hedgehog pathway in medulloblastoma. *Proceedings of the National Academy of Sciences of the United States of America*.

[54] Alva J. A., Lee G. E., Escobar E. E., Pyle A. D. (2011). Phosphatase and tensin homolog regulates the pluripotent state and lineage fate choice in human embryonic stem cells. *STEM CELLS*.

[55] Dahia P. L. M. (2000). PTEN, a unique tumor suppressor gene. *Endocrine-Related Cancer*.

[56] Gewurz B. E., Mar J. C., Padi M. (2011). Canonical NF-*κ*B activation is essential for Epstein-Barr virus latent membrane protein 1 TES2/CTAR2 gene regulation. *Journal of Virology*.

[57] Annerén C. (2008). Tyrosine kinase signalling in embryonic stem cells. *Clinical Science*.

[58] Choi J., Lee S., Mallard W. (2015). A comparison of genetically matched cell lines reveals the equivalence of human iPSCs and ESCs. *Nature Biotechnology*.

[59] Rickinson A., Kieff E., Howley P., Knipe D. (2007). Epstein-Barr virus. *Fields Virology*.

[60] Izumi K. M., Kaye K. M., Kieff E. D. (1997). The Epstein-Barr virus LMP1 amino acid sequence that engages tumor necrosis factor receptor associated factors is critical for primary B lymphocyte growth transformation. *Proceedings of the National Academy of Sciences of the United States of America*.

[61] Izumi K. M., Kieff E. D. (1997). The Epstein-Barr virus oncogene product latent membrane protein 1 engages the tumor necrosis factor receptor-associated death domain protein to mediate B lymphocyte growth transformation and activate NF-*κ*B. *Proceedings of the National Academy of Sciences of the United States of America*.

[62] Izumi K. M., McFarland E. C., Riley E. A., Rizzo D., Chen Y., Kieff E. (1999). The residues between the two transformation effector sites of Epstein-Barr virus latent membrane protein 1 are not critical for B-lymphocyte growth transformation. *Journal of Virology*.

[63] Soni V., Cahir-McFarland E., Kieff E. (2007). LMP1 TRAFficking activates growth and survival pathways. *Advances in Experimental Medicine and Biology*.

[64] Li J., Wang G., Wang C. (2007). MEK/ERK signaling contributes to the maintenance of human embryonic stem cell self-renewal. *Differentiation*.

[65] Zhang X., Yalcin S., Lee D.-F. (2011). FOXO1 is an essential regulator of pluripotency in human embryonic stem cells. *Nature Cell Biology*.

[66] Park I.-H., Zhao R., West J. A. (2008). Reprogramming of human somatic cells to pluripotency with defined factors. *Nature*.

[67] Niwa H., Miyazaki J.-I., Smith A. G. (2000). Quantitative expression of Oct-3/4 defines differentiation, dedifferentiation or self-renewal of ES cells. *Nature Genetics*.

[68] Fong H., Hohenstein K. A., Donovan P. J. (2008). Regulation of self-renewal and pluripotency by Sox2 in human embryonic stem cells. *Stem Cells*.

[69] Chambers I., Colby D., Robertson M. (2003). Functional expression cloning of Nanog, a pluripotency sustaining factor in embryonic stem cells. *Cell*.

[70] Chambers I., Silva J., Colby D. (2007). Nanog safeguards pluripotency and mediates germline development. *Nature*.

[71] Amit M., Carpenter M. K., Inokuma M. S. (2000). Clonally derived human embryonic stem cell lines maintain pluripotency and proliferative potential for prolonged periods of culture. *Developmental Biology*.

[72] Wang G., Zhang H., Zhao Y. (2005). Noggin and bFGF cooperate to maintain the pluripotency of human embryonic stem cells in the absence of feeder layers. *Biochemical and Biophysical Research Communications*.

[73] Xu C., Rosler E., Jiang J. (2005). Basic fibroblast growth factor supports undifferentiated human embryonic stem cell growth without conditioned medium. *Stem Cells*.

[74] Xu R.-H., Peck R. M., Li D. S., Feng X., Ludwig T., Thomson J. A. (2005). Basic FGF and suppression of BMP signaling sustain undifferentiated proliferation of human ES cells. *Nature Methods*.

[75] Levenstein M. E., Ludwig T. E., Xu R.-H. (2006). Basic fibroblast growth factor support of human embryonic stem cell self-renewal. *STEM CELLS*.

[76] James D., Levine A. J., Besser D., Hemmati-Brivanlou A. (2005). TGF*β*/activin/nodal signaling is necessary for the maintenance of pluripotency in human embryonic stem cells. *Development*.

[77] Saha S., Ji L., De Pablo J. J., Palecek S. P. (2008). TGF*β*/activin/nodal pathway in inhibition of human embryonic stem cell differentiation by mechanical strain. *Biophysical Journal*.

[78] Yu J., Thomson J. A. (2008). Pluripotent stem cell lines. *Genes and Development*.

[79] Fink S. P., Mikkola D., Willson J. K. V., Markowitz S. (2003). TGF-*β*-induced nuclear localization of Smad2 and Smad3 in Smad4 null cancer cell lines. *Oncogene*.

[80] Sun Z., Jin P., Tian T., Gu Y., Chen Y.-G., Meng A. (2006). Activation and roles of ALK4/ALK7-mediated maternal TGF*β* signals in zebrafish embryo. *Biochemical and Biophysical Research Communications*.

[81] Banas M. C., Parks W. T., Hudkins K. L. (2007). Localization of TGF-*β* signaling intermediates Smad2, 3, 4, and 7 in developing and mature human and mouse kidney. *Journal of Histochemistry and Cytochemistry*.

[82] Mancino M., Strizzi L., Wechselberger C. (2008). Regulation of human Cripto-1 gene expression by TGF-*β*1 and BMP-4 in embryonal and colon cancer cells. *Journal of Cellular Physiology*.

[83] Xu R.-H., Sampsell-Barron T. L., Gu F. (2008). NANOG is a direct target of TGF*β*/activin-mediated SMAD signaling in human ESCs. *Cell Stem Cell*.

[84] Hayashi H., Abdollah S., Qiu Y. (1997). The MAD-related protein Smad7 associates with the TGF*β* receptor and functions as an antagonist of TGF*β* signaling. *Cell*.

[85] Imamura T., Takase M., Nishihara A. (1997). Smad6 inhibits signalling by the TGF-*β* superfamily. *Nature*.

[86] Nakao A., Afrakhte M., Morén A. (1997). Identification of Smad7, a TGF*β*-inducible antagonist of TGF-*β* signalling. *Nature*.

[87] Wood V. H. J., O'Neil J. D., Wei W., Stewart S. E., Dawson C. W., Young L. S. (2007). Epstein-Barr virus-encoded EBNA1 regulates cellular gene transcription and modulates the STAT1 and TGF*β* signaling pathways. *Oncogene*.

[88] Siegel P. M., Massagué J. (2003). Cytostatic and apoptotic actions of TGF-*β* in homeostasis and cancer. *Nature Reviews Cancer*.

[89] Chen Y. G., Meng A. M. (2004). Negative regulation of TGF-*β* signaling in development. *Cell Research*.

[90] Kang H. B., Kim J. S., Kwon H.-J. (2005). Basic fibroblast growth factor activates ERK and induces c-Fos in human embryonic stem cell line MizhES1. *Stem Cells and Development*.

[91] Vallier L., Alexander M., Pedersen R. A. (2005). Activin/Nodal and FGF pathways cooperate to maintain pluripotency of human embryonic stem cells. *Journal of Cell Science*.

[92] Sakanaka C., Sun T., Williams L. T. (2000). New steps in the Wnt/*β*-catenin signal transduction pathway. *Recent Progress in Hormone Research*.

[93] Miki T., Yasuda S.-Y., Kahn M. (2011). Wnt/*β*-catenin signaling in embryonic stem cell self-renewal and somatic cell reprogramming. *Stem Cell Reviews and Reports*.

[94] Pébay A., Wong R. C. B., Pitson S. M. (2005). Essential roles of sphingosine-1-phosphate and platelet-derived growth factor in the maintenance of human embryonic stem cells. *Stem Cells*.

[95] Soldner F., Laganière J., Cheng A. W. (2011). Generation of isogenic pluripotent stem cells differing exclusively at two early onset parkinson point mutations. *Cell*.

[96] Byers B., Cord B., Nguyen H. N. (2011). SNCA triplication parkinson's patient's iPSC-Derived DA neurons accumulate *α*-synuclein and are susceptible to oxidative stress. *PLoS ONE*.

[97] Nguyen H. N., Byers B., Cord B. (2011). LRRK2 mutant iPSC-derived da neurons demonstrate increased susceptibility to oxidative stress. *Cell Stem Cell*.

[98] Imaizumi Y., Okada Y., Akamatsu W. (2012). Mitochondrial dysfunction associated with increased oxidative stress and *α*-synuclein accumulation in PARK2 iPSC-derived neurons and postmortem brain tissue. *Molecular Brain*.

[99] Sánchez-Danés A., Richaud-Patin Y., Carballo-Carbajal I. (2012). Disease-specific phenotypes in dopamine neurons from human iPS-based models of genetic and sporadic Parkinson's disease. *EMBO Molecular Medicine*.

[100] Ryan S. D., Dolatabadi N., Chan S. F. (2013). Isogenic human iPSC Parkinson's model shows nitrosative stress-induced dysfunction in MEF2-PGC1alpha transcription. *Cell*.

[101] Miller J. D., Ganat Y. M., Kishinevsky S. (2013). Human iPSC-based modeling of late-onset disease via progerin-induced aging. *Cell Stem Cell*.

[102] Reinhardt P., Schmid B., Burbulla L. F. (2013). Genetic correction of a lrrk2 mutation in human iPSCs links parkinsonian neurodegeneration to ERK-dependent changes in gene expression. *Cell Stem Cell*.

[103] Sanders L. H., Laganière J., Cooper O. (2014). LRRK2 mutations cause mitochondrial DNA damage in iPSC-derived neural cells from Parkinson's disease patients: reversal by gene correction. *Neurobiology of Disease*.

[104] Schöndorf D. C., Aureli M., McAllister F. E. (2014). IPSC-derived neurons from GBA1-associated Parkinson's disease patients show autophagic defects and impaired calcium homeostasis. *Nature Communications*.

